# Retracted: Prevalence of Bacteriuria and Antimicrobial Susceptibility Patterns among Diabetic and Nondiabetic Patients Attending at Debre Tabor Hospital, Northwest Ethiopia

**DOI:** 10.1155/2018/8964958

**Published:** 2018-06-06

**Authors:** International Journal of Microbiology

At the request of the authors, the article titled “Prevalence of Bacteriuria and Antimicrobial Susceptibility Patterns among Diabetic and Nondiabetic Patients Attending at Debre Tabor Hospital, Northwest Ethiopia” [1] has been retracted. The article was found to contain a substantial amount of material from a previously unpublished thesis.

The first author Seble Worku takes the full responsibility and apologizes for the error.
